# Network Analysis of the CSF Proteome Characterizes Convergent Pathways of Cellular Dysfunction in ALS

**DOI:** 10.3389/fnins.2021.642324

**Published:** 2021-03-17

**Authors:** Alexander G. Thompson, Elizabeth Gray, Philip D. Charles, Michele T. M. Hu, Kevin Talbot, Roman Fischer, Benedikt M. Kessler, Martin R. Turner

**Affiliations:** ^1^Nuffield Department of Clinical Neurosciences, University of Oxford, Oxford, United Kingdom; ^2^Target Discovery Institute, Centre for Medicines Discovery, Nuffield Department of Medicine, University of Oxford, Oxford, United Kingdom

**Keywords:** cerebrospinal fluid, amyotrophic lateral sclerosis, motor neuron disease, biomarker, proteomics, proteomics & bioinformatics, WGCNA, network analysis

## Abstract

**Background:**

Amyotrophic lateral sclerosis is a clinical syndrome with complex biological determinants, but which in most cases is characterized by TDP-43 pathology. The identification in CSF of a protein signature of TDP-43 network dysfunction would have the potential to inform the identification of new biomarkers and therapeutic targets.

**Methods:**

We compared CSF proteomic data from patients with ALS (*n* = 41), Parkinson’s disease (*n* = 19) and healthy control participants (*n* = 20). Weighted correlation network analysis was used to identify modules within the CSF protein network and combined with gene ontology enrichment analysis to functionally annotate module proteins. Analysis of module eigenproteins and differential correlation analysis of the CSF protein network was used to compare ALS and Parkinson’s disease protein co-correlation with healthy controls. In order to monitor temporal changes in the CSF proteome, we performed longitudinal analysis of the CSF proteome in a subset of ALS patients.

**Results:**

Weighted correlation network analysis identified 10 modules, including those enriched for terms involved in gene expression including nucleic acid binding, RNA metabolism and translation; humoral immune system function, including complement pathways; membrane proteins, axonal outgrowth and adherence; and glutamatergic synapses. Immune system module eigenproteins were increased in ALS, whilst axonal module eigenproteins were decreased in ALS. The 19 altered protein correlations in ALS were enriched for gene expression (OR 3.05, *p* = 0.017) and membrane protein modules (OR 17.48, *p* = 0.011), including intramodular hub proteins previously identified as TDP-43 interactors. Proteins decreasing over longitudinal analysis ALS were enriched in glutamatergic synapse and axonal outgrowth modules. Protein correlation network disruptions in Parkinson’s disease showed no module enrichment.

**Conclusions:**

Alterations in the co-correlation network in CSF samples identified a set of pathways known to be associated with TDP-43 dysfunction in the pathogenesis of ALS, with important implications for therapeutic targeting and biomarker development.

## Introduction

Amyotrophic lateral sclerosis (ALS) is a fatal neurodegenerative disease, associated with selective loss of motor neurons in the spinal cord and brain. Alterations in multiple cellular pathways have been implicated in the pathogenesis of ALS, including excitotoxicity, cellular energy metabolism, protein degradation and non-cell autonomous glial mechanisms, representing multiple overlapping tributaries into the final common pathway of motor neuron degeneration (Talbot et al., 2018). Since the discovery of mislocalized cytoplasmic aggregates of 43 kDa trans-active response DNA-binding protein (TDP-43) as the neuropathological hallmark of nearly all ALS cases (Neumann et al., 2006), focus has fallen on mechanisms related to alterations in the function and behavior of TDP-43, particularly its roles in RNA splicing, the stress response and its propensity for aggregation (Taylor et al., 2016).

Evidence of perturbations in many pathways implicated in ALS have been identified in biofluid samples from ALS patients. Alterations in markers of oxidative stress, glial and immune activation, axonal degeneration and protein degradation mechanisms have been detected in patient samples using candidate-driven and untargeted studies of cerebrospinal fluid (CSF) proteins and metabolites (Turner et al., 2009).

A major advantage of the high-dimensional data produced by untargeted approaches is the capability to explore co-ordinated network alterations, engendering broader understanding of the pathophysiological changes associated with a disease or phenotype. Analytical techniques based on co-correlation, such as weighted gene correlation network analysis (WGCNA) (Langfelder and Horvath, 2008) and differential gene correlation analysis (McKenzie et al., 2016) have been applied widely in genomics and proteomics to derive regulatory networks, understand disease-associated alterations in protein networks and identify candidate therapeutic targets. Here, we apply this approach to CSF, comparing network changes in patients with ALS with healthy controls and, in order to distinguish disease-specific changes from neurodegeneration-associated changes, patients with Parkinson’s disease (PD) aiming to identify network disruption overlooked by conventional analysis.

## Materials and Methods

### Participants and Sampling

Ethical approval for this study was obtained from South Central Oxford Ethics Committee B (08/H0605/85) NRES Central Committee South Central – Berkshire (14/SC/0083 and 10/H0505/71). All participants provided written consent (or gave permission for a carer to sign on their behalf). The study included 43 patients with ALS, 20 patients with Parkinson’s disease, and 20 healthy control subjects. Patients with ALS were recruited from the Oxford ALS Centre, Oxford, United Kingdom and patients with Parkinson’s disease were recruited through the Oxford Parkinson’s Disease Centre, Oxford, United Kingdom.

CSF was collected at baseline and, in ALS patients, every 6 months when available. Clinical data was ascertained on the same day. CSF samples were processed in accordance with consensus guidelines for biomarker development within 1 h of sampling and stored at −80°C until use. Symptom onset was defined as first weakness reported by patients. Disease progression rate was calculated per-visit using the revised ALS functional rating scale (ALSFRS-R) by [48 – ALSFRS-R]/[months from symptom onset].

### Proteomic Analysis

The raw data used in this analysis has been previously published (Thompson et al., 2018b). In brief, samples of CSF were thawed on ice and digested using heat stable immobilized trypsin as per the manufacturer’s instructions (SMART digest, Thermo Fisher Scientific, United Kingdom). 50 μL of CSF was mixed with 150 μL SMART digest buffer and added to SMART digest plates. Samples were incubated at 70°C with shaking at 1,400 rpm for 60 min. Digested samples were desalted using SOLAμ plates and dried by vacuum centrifugation. Samples were resuspended in 20 μL buffer A (2% acetonitrile, 0.1% formic acid in water) and kept at −20°C until analysis. Peptide concentrations were assayed using a Pierce quantitative colorimetric peptide assay (Thermo Fisher Scientific, United Kingdom) according to the manufacturer’s instructions. A pooled sample was produced by combining equal quantities of digested peptide from each individual sample and injected after every tenth sample for use in quality control analysis.

Peptides were analyzed by nano ultra-high performance liquid chromatography tandem mass spectrometry (nUHPLC LC-MS/MS) using a Dionex Ultimate 3000 UHPLC (Thermo Fisher Scientific, Germany) coupled to a Q Exactive HF tandem mass spectrometer (Thermo Fisher Scientific, Germany). 500 nL of peptides from each sample were injected and analyzed using a 60-min linear gradient at a 250 nL/min flow rate. The gradient used to elute the peptides started at 3 min with 2% buffer B (0.1% TFA and 5% DMSO in CH_3_CN) increasing to 5% by 6 min followed by an increase up to 35% by 63 min. The data were acquired with a resolution of 60,000 full-width at half maximum ion intensity with a mass/charge ratio of 400 and a lock mass enabled at 445.120025 m/z. The 12 most abundant precursor ions in each MS1 scan were selected for fragmentation by higher-energy collisional dissociation (HCD) at a normalized collision energy of 28 followed by exclusion for 27 s.

Raw MS data were analyzed using Progenesis QI for Proteomics software v3.0 (Non-linear Dynamics). MS/MS spectra were searched against the UniProt Homo Sapiens Reference proteome (retrieved 01/06/2017) using Mascot v2.5.1 (Matrix Science) allowing for a precursor mass tolerance of 10 ppm and a fragment ion tolerance of 0.05 Da. Deamidation on asparagine and glutamine and oxidation on methionine were included as variable modifications. The peptide false discovery rate (FDR) was set at 1% and all peptides with an ion score higher than 20 into were imported into Progenesis QIP. Proteins that were defined with at least one unique peptide were included in the protein data set for further analysis (289 proteins had one unique peptide; Supplementary Figure 1). Protein abundance values were centered to a background median (similar to the Progenesis QIP ‘robust mean’ used for normalization within the software), where the background was taken as the 90% of proteins with the lowest variance across all runs (Keilhauer et al., 2015). Values were then scaled by median absolute deviation.

### Statistical Analysis

Statistical and bioinformatic analysis was performed in R version 4.0.2. Correction for multiple comparisons was performed using the Benjamini-Hochberg step-up procedure, with adjusted *p* < 0.1 taken to indicate statistical significance. Raw, uncorrected *p*-values were reported where fewer than 20 hypothesis tests were carried out, using *p* < 0.05 to denote statistical significance.

### Weighted Correlation Network Analysis

Weighted correlation network analysis was performed with the weighted gene correlation network analysis (WGCNA) package in R. Three outlying samples (two ALS and one Parkinson’s disease) were identified using hierarchical clustering and were excluded from subsequent analysis (Supplementary Figure 2; participant demographics including longitudinal sampling Table 1). Eighteen proteins were excluded due to an excessive degree of missing data (>50% from any group). Only baseline samples visits for longitudinal participants were included in network analysis. A signed, weighted network was constructed using soft thresholding power = 7 using Pearson correlation as the dissimilarity measure, minimum module size 5 and cut height 0.05. The most highly connected 10% of proteins within each module (highest *k*_in_) were denoted intramodular hub proteins. Module stability was assessed by iterating network construction using the same settings, randomly excluding one sample from each run and comparing the proportion of shared protein module assignments between with the reference network. Network graphs were produced in R using the igraph package.

**TABLE 1:** Baseline demographic features of participants included in WGCNA analysis.

	ALS	HC	PD	*p*
n, visit 1	41	20	19	–
n, visit 2	20	–	–	–
n, visit 3	12	–	–	–
n, visit 4	10	–	–	–
n, visit 5	2	–	–	–
Age at sampling, years (mean ± SD)	62.62 ± 9.99	58.53 ± 8.57	62.87 ± 3.95	0.263*
Age at symptom onset, years (mean ± SD)	59.95 ± 10.75	–	61.12 ± 3.87	0.925*
Male participants, *n* (%)	30 (73.2)	11 (55)	10 (52.6)	0.193^+^
Baseline disease progression rate, points/month (median [IQR])	0.5 [0.27–1.00]	–	–	–

*Kruskal-Wallis *H* test.

*^+^Fisher Exact test.*

*ALS, amyotrophic lateral sclerosis; HC, healthy control; PD, Parkinson’s disease; SD, standard deviation; IQR, interquartile range.*

Module-phenotype associations were analyzed by comparing module eigenprotein expression between conditions with a pairwise Mann-Whitney *U* test, comparing healthy controls with ALS or PD samples.

### Comparisons With ALS-FTD Cortical Networks

The CSF protein network was compared with a previously published frontal cortex proteomic dataset from control, ALS, FTD and ALS-FTD patients using a cross-tabulation approach (Umoh et al., 2018). Individual module protein and gene assignments were compared between CSF and frontal cortex module allocations for each module pair using a hypergeometric test.

### Differential Correlation Analysis

Analysis of differential correlation were performed by within-group pairwise Pearson correlation of protein abundance in healthy control, ALS and PD samples and correlations compared using Fisher’s *r*-to-*z* transformation. Resulting p-values were corrected for multiple comparisons using the Benjamini-Hochberg step-up procedure.

### Enrichment Analysis

Proteins were abstracted to genes for gene ontology (GO) and module enrichment analysis. GO enrichment analysis was performed in R with TopGo using the “weight” algorithm. Foreground lists comprised genes within each module or differentially correlated proteins, the background list comprised all genes identified in the proteomic analysis. Module enrichment analysis was performed using a hypergeometric test.

### Longitudinal Analysis

Longitudinal analysis was performed in R using the nlme package. Models were constructed using log_–_transformed longitudinal data, including only participants for whom longitudinal samples were available. Individual participants were specified as random effects and anchored to the date of the initial visit using linear mixed effects modeling with a random intercept, fixed slope model, uncorrelated covariance structure and degrees of freedom as calculated by Pinheiro and Bates (Pinehiro and Bates, 2000).

## Results

### The CSF Protein Correlation Network

**FIGURE 1:**
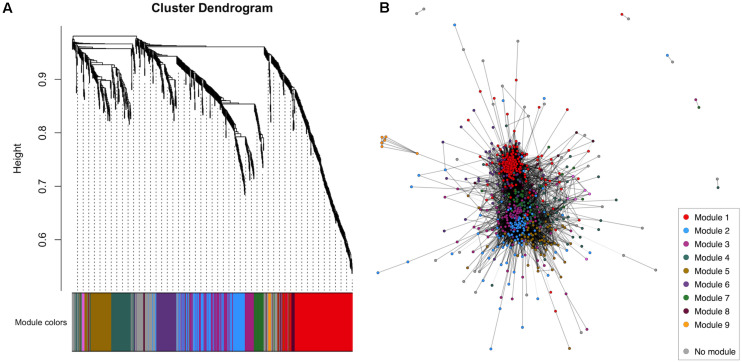
WGCNA of the healthy control CSF proteome. **(A)** Cluster dendrogram indicating module allocation. **(B)** Network graph indicating modules. For ease of visualization, pairwise correlations with FDR-adjusted *p* > 0.01 have been excluded from this graph. CSF, cerebrospinal fluid; WGCNA, weighted gene correlation network analysis.

**FIGURE 2:**
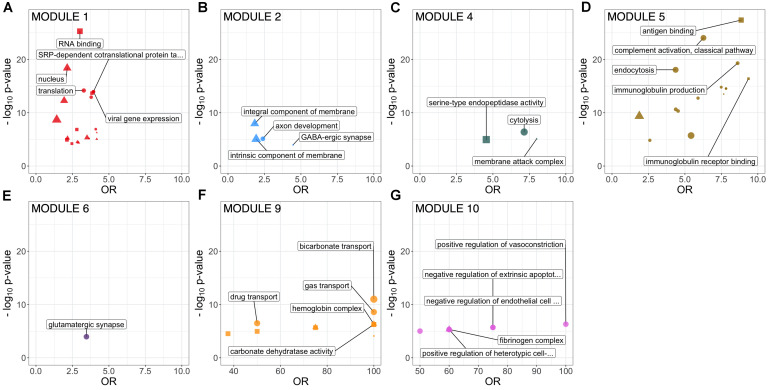
GO term enrichment of identified CSF protein network modules (FDR-adjusted *p* < 0.1). Size proportional to number of annotated proteins within a GO term in that module. Top 5 GO terms by *p*-value are labeled. All significantly enriched GO terms are detailed in Supplementary Table 1. CSF, cerebrospinal fluid; GO, Gene Ontology.

WGCNA of the CSF proteome yielded a protein network comprising 776 proteins in 10 modules ranging from 7 to 183 proteins (Figure 1). 107 proteins were not allocated to a module. To understand the biological relevance of the protein correlation network modules, Gene Ontology (GO) enrichment analysis was performed (Figure 2 and Supplementary Table 1).

Two large modules demonstrated significant enrichment for distinct groups of GO terms. Module 1, the largest comprising 183 proteins, was enriched in intracellular proteins annotated to cytoplasmic and nuclear intracellular compartments. Concordant with this, module one proteins were enriched for functions involved in gene expression including nucleic acid binding, RNA metabolism and translation (Figure 2 and Supplementary Table 1). Module 2, comprising 115 proteins was enriched in GO terms relating to axon development, neurons, GABAergic synapses and the cell membrane. Module 4 (75 proteins) was enriched for cytolysis and the membrane attack complex. Module 5 (67 proteins) was enriched in immune system proteins relating primarily to the humoral immune system including immunoglobulins and complement, B-cell signaling and fibrinolysis. Smaller modules were enriched in glutamatergic synapse proteins (module 6, 58 proteins); blood proteins involved in gas transport (module 9, 7 proteins); fibrinogen complex, peptide hormone secretion and vasoconstriction (module 10, 7 proteins). Module stability analysis indicated reproducible protein-module assignment for >75% of proteins in over 50% of iterations for modules 1, 2, 4, 5, and 6, and >50% for module 3 (Supplementary Figure 3).

**FIGURE 3:**
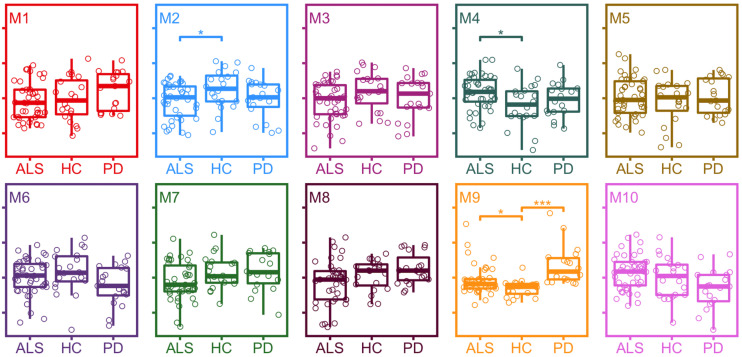
Expression of module eigenproteins between conditions. *p < 0.05, **p < 0.01, ***p < 0.001. ALS, amyotrophic lateral sclerosis; HC, healthy control; PD, Parkinson’s disease.

Differences in module protein expression as measured by module eigenproteins were observed between ALS and healthy control samples for module 2 (healthy control median 0.054, ALS median 0.001, *p* = 0.031), module 4 (healthy control median −0.036, ALS median 0.036, *p* = 0.016), and module 9 (healthy control median −0.052, ALS median −0.036, *p* = 0.015) and between PD and healthy control samples for module 9 (healthy control median −0.052, PD median 0.053, *p* < 0.001; Figure 3).

### Differential Protein Correlation Analysis Reveals Altered Cellular Processes in ALS

**FIGURE 4:**
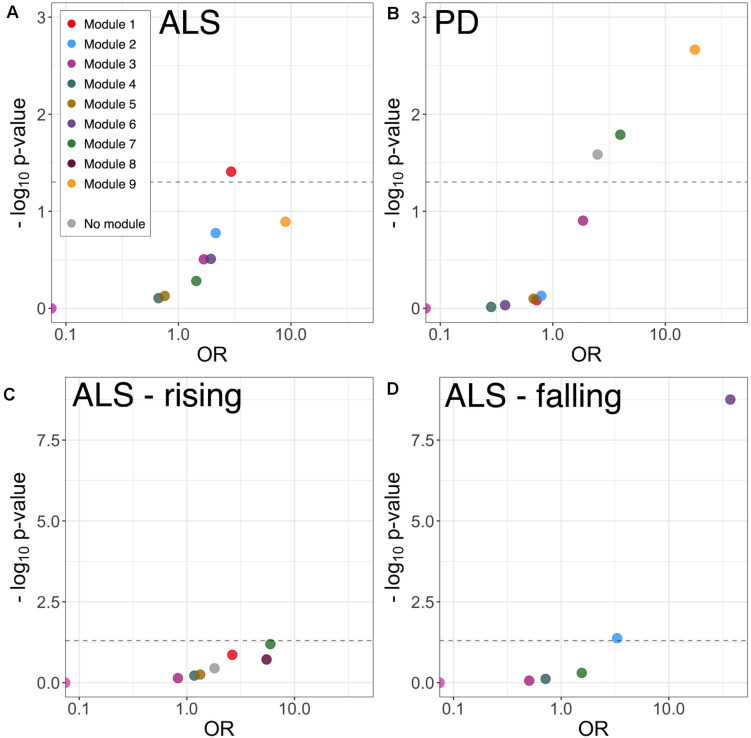
Module enrichment of differentially correlated proteins in ALS **(A)** and PD **(B)**, and longitudinally falling **(C)** or rising **(D)** proteins in ALS. ALS, amyotrophic lateral sclerosis; PD, Parkinson’s disease.

To examine disease-related disruptions in the protein correlation network at a more granular level, differential correlation analysis was performed, comparing pairwise protein correlations in CSF from ALS and PD patients with those in healthy control CSF. This identified 11 significantly altered correlations between 19 proteins (19 genes) in ALS (Supplementary Table 2). There was no significant GO term enrichment (false discovery rate (FDR)-adjusted *p* < 0.1) amongst differentially correlated proteins, likely attributable to the small number of proteins in the foreground list. There was enrichment of proteins in module 1 (9/19 proteins, OR 3.05, *p* = 0.017) and module 9 (2/19 proteins, OR 17.48, *p* = 0.011; Figure 4A).

Module 1 proteins with altered correlation in ALS included RNA and DNA binding proteins and proteins involved in transcription and translation: Putative elongation factor 1-alpha 1 (EEF1A1), Histone H2B type 1-N (H2BC11), Acidic leucine-rich nuclear phosphoprotein 32 family member A (ANP32A) and Y-box-binding protein 1 (YBX1); the microtubule protein Tubulin beta chain (TUBB); the glycolytic enzymes Glyceraldehyde-3-phosphate dehydrogenase (GAPDH) and lactate dehydrogenase (LDHA); and Macrophage migration inhibitory factor (MIF). Three of the Module 1 proteins with altered correlation in ALS were intramodular hub proteins (EEF1A1, H2BC11 and GAPDH).

There were three altered correlations in which both proteins were within module 1: H2BC11 with TUBB (*r* = 0.97 HC, 0.56 ALS, FDR-adjusted *p* = 0.035), EEFA1A with TUBB (*r* = 0.97 HC, 0.57 ALS, *p* = 0.057), and GAPDH with MIF (*r* = 0.93 HC, 0.25 ALS, *p* = 0.057).

Differential correlation analysis comparing PD and healthy controls identified 27 significant altered correlations between 36 proteins (36 genes). No significant GO enrichment was identified. Dyscorrelated proteins were enriched in module 7 (5/36 proteins, OR 3.98, *p* = 0.016) and module 9 proteins (3/36 proteins, OR 18.31, *p* = 0.002) including blood proteins and proteins involved in adhesion and carbohydrate metabolism (Figure 4B and Supplementary Table 2).

### Longitudinal Analysis Indicates Modulation in Axon Guidance and Neurodevelopment Pathways in ALS

Linear mixed-model analysis identified 10 longitudinally increasing and 15 longitudinally decreasing proteins in ALS patients (FDR-adjusted *p* < 0.1; Supplementary Table 3). The proteins with longitudinally increasing abundance comprised proteins present at high levels in plasma including complement components C7 and C1S, Thyroxine-binding globulin, and immunoglobulins; and extracellular matrix proteins Laminin subunit alpha-2 and Galectin-3-binding protein. There was no significant GO or module enrichment of increasing proteins (Figure 4C).

Proteins with longitudinally decreasing abundance were enriched in module 2 proteins (Figure 4D, enriched for membrane, neuronal cell body and axon development 5/15 proteins; OR 3.31, *p* = 0.042) and module 6 proteins (glutamatergic synapse; 10/15 proteins OR 37.21, *p* < 0.001). Though lacking significant GO term enrichment, they were annotated to concordant, disease-relevant GO terms. These included axonal guidance and neurodevelopment (Neurofascin, Semaphorin-7A, Ciliary neurotrophic factor receptor subunit alpha, Peptidyl-glycine alpha-amidating monooxygenase, Neuritin, Disintegrin and metalloproteinase domain-containing protein 22), synapse assembly and function (Calsyntenin-3, Receptor-type tyrosine-protein phosphatase-like N, Neurofascin), neuropeptide signaling [Neuroendocrine protein 7B2, identified as a candidate ALS biomarker in a previous CSF proteomic study (Ranganathan et al., 2005)] and RNA processing (ATP-dependent RNA helicase DHX8). Of the longitudinally decreasing proteins, Neuritin and Neurofascin were intramodular hubs.

### Frontal Cortex and CSF Protein Networks Show Major Differences

**FIGURE 5:**
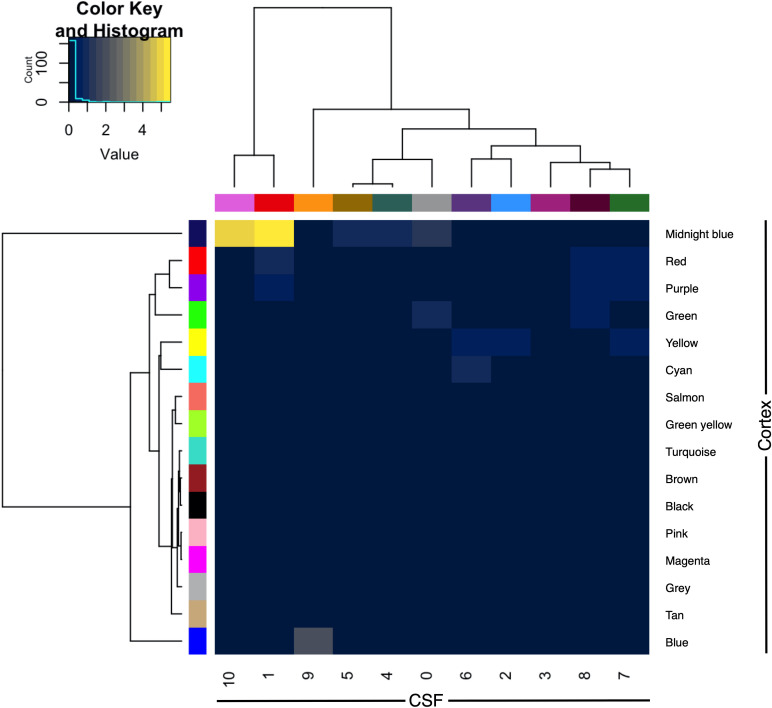
Module preservation of CSF (columns) and frontal cortex (rows) by cross-tabulation. Significant overlaps were observed between CSF modules 1 and 10 with frontal cortex module 9 (midnight blue). Color indicates –log_10_ *p*-value for enrichment. CSF, cerebrospinal fluid.

The CSF protein network was compared with that of a previously published frontal cortex protein network derived from shotgun proteomic analysis of control, ALS, FTD and ALS-FTD patient tissue. The overlap between proteins and genes between the two datasets was limited (intersect 107 proteins of 776 CSF and 2612 cortex; intersect 283 genes of 684 CSF and 2487 cortex genes). Module preservation analysis by cross tabulation (pairwise enrichment analysis of CSF and frontal cortex modules) demonstrated no evidence of preservation of any modules. When abstracted to genes, there was significant, albeit modest, overlap of frontal cortex module 9 (midnight blue) with CSF module 1 (9/170, OR 10.52, *p* < 0.001) and CSF module 10 (3/6, OR 135.95, *p* < 0.001; Figure 5).

## Discussion

This study analyzed a large CSF proteomic dataset to delineate the overall protein network structure in healthy controls, ALS and PD. The analysis identified several major protein modules, the first enriched in intracellular compartment proteins and functions involved in gene expression and regulation. The second large module, was enriched with proteins involved in axonal development, inhibitory synapses and membrane proteins. Smaller, less stable, modules were enriched for immune system proteins (modules 4 and 5), glutamatergic synapse proteins (module 6) and blood proteins involved in gas transport (module 9), endothelial and clotting pathways (module 10).

Module eigenprotein-phenotype relationships identified decreased expression of module 2 and increased expression of modules 4 and 9 in ALS, and of module 9 in PD. Module 2 proteins include neural growth factors, guidance proteins and cell adhesion molecules, many of which have been studied in ALS and FTD as biomarker candidates. The module 2 intramodular hub protein Ephrin type A receptor 4 has been identified as a modifier of ALS severity, with lower levels associated with later onset and more rapid disease progression (Van Hoecke et al., 2012). Missense mutations in CDH13, encoding Cadherin 13 precursor, another module 2 intramodular hub protein, have been identified in sporadic ALS patients, though this finding has not been replicated (Daoud et al., 2011). Altered regulation of synaptic adhesion proteins in module 2 Neurexin 1 and Neurexin 3 (of which Neurexin 1 is a module 2 intramodular hub protein) have also been identified as a consequence of TDP-43 depletion (Polymenidou et al., 2011).

The finding of decreases in module 2 synaptic proteins in ALS is consistent with previous work in ALS, but differs from Alzheimer’s disease, in which increases in levels of synaptic proteins in CSF have been observed (Dayon et al., 2018; Portelius et al., 2018; Higginbotham et al., 2020). It is possible that the low levels observed in ALS reflect synaptic loss, whilst in Alzheimer’s they indicate an active process within synapses and alterations in synaptic protein turnover (Hark et al., 2021).

Module 4 contains proteins involved in the innate immune response including the ALS microglial activity marker Chitotriosidase 1 (Steinacker et al., 2018; Thompson et al., 2018b, 2019; Vu et al., 2020), as well as complement components and apolipoproteins. Marked inflammatory change, particularly involving microglia and involving complement, is a well-described feature of ALS neuropathology (Brettschneider et al., 2012; Bahia El Idrissi et al., 2016), whilst alterations in apolipoprotein metabolism have been implicated in the development of ALS and as a modulator of disease progression (Mariosa et al., 2017; Ingre et al., 2020). Alterations in module 9 may be a reflection of altered blood-brain or blood-CSF barrier function (Garbuzova-Davis and Sanberg, 2014), though this is less well-recognized as a feature of PD (Desai et al., 2007).

Differential protein correlation analysis provided evidence of disease-specific alterations in relevant network modules. Several of the proteins with altered correlation derive from pathways strongly implicated in ALS pathogenesis. In particular, alterations in gene expression pathways have been demonstrated in disease models and *post mortem* tissue from ALS patients (Polymenidou et al., 2011; Krach et al., 2018). Differential correlations in ALS were identified in H2BC11, a histone protein, YBX1, a transcription factor implicated in ALS through model and *post mortem* tissue analysis, identified as an interactor of TDP-43 and stress granule component (Anders et al., 2018; Nijssen et al., 2018; Feneberg et al., 2020; La Cognata et al., 2020), and EEF1A1, a translational elongation factor and, like YBX1, stress granule component and TDP-43 interactor (Kim et al., 2010; Anders et al., 2018). EEF1A1 and YBX1 are also components of the synaptic protein expression machinery, potentially linking alterations in module 2 protein levels with loss of correlation in module 1 (Holt et al., 2019). TUBB, again a TDP-43 interactor (Freibaum et al., 2010), dimerizes with Tubulin alpha to form microtubules; mutations in genes encoding cytoskeletal proteins including Tubulin alpha (though not TUBB) have been identified as a rare cause of ALS (Smith et al., 2014). Alterations were also observed in the relationship of several enzymes, such as GAPDH, involved in carbohydrate metabolism, implicated through disease models and epidemiological studies (Kioumourtzoglou et al., 2015; Szelechowski et al., 2018).

The main signal emerging from longitudinal analysis indicated striking progressive downregulation of proteins in the module enriched for glutamatergic synapse proteins as well as axonal and neuronal proteins. This is in keeping with the progressive loss of axons, neurons and synapses that are a core pathological feature of ALS (Sasaki and Maruyama, 1994).

Analysis incorporating comparing the CSF protein network with a previously published frontal cortex protein correlation network indicated limited topological overlap between this CSF protein network and that of frontal cortex (Umoh et al., 2018).

Despite the lack of topological overlap, there was similarity in the functional annotation of identified modules in frontal cortex and CSF, notably between CSF module 1 and frontal cortex module 2, both enriched in transcription and translation-related ontological terms. CSF module 5 and cortex module 15 were enriched in antigen binding and immune system terms, whilst synaptic, membrane and axons terms were identified in cortex module 1 overlapping with CSF module 2 and module 6 (specifically glutamatergic synapse in the latter).

Although the CSF proteome receives a significant contribution from the brain, much of this arises from the white matter and gray matter regions beyond the frontal cortex. In addition, a large proportion of the CSF protein constitution arises through filtration of blood and secretion from the choroid plexus and includes a large proportion of classically secreted and non-classically secreted proteins (Thompson et al., 2018a). Furthermore, many neuronal and glial intracellular proteins might not be translocated into the extracellular space and hence the CSF in normal conditions, and the egress of proteins from CSF if determined by additional physiological processes (such as CSF flow rate) that would not necessarily affect all proteins proportionately (Reiber, 2001). The relatively limited overlap in the protein identifications, likely attributable to differences in methodological approach and the challenges of achieving proteomic depth in biological fluids, is also a consideration.

There are several limitations to this study. Genotype data, including presence of the ALS-causing *C9orf72* hexanucleotide repeat expansion, was not included since testing was not widely available at the time of sampling of participants in the study. Though sharing the main pathological features of sporadic ALS, *C9orf72* genotype could influence CSF network structure, but in this sporadic cohort it would not be expected to assert major effects, though would potentially have provided insights into the molecular divergence of genetic and non-genetic ALS. A significant proportion of proteins were identified based on one unique peptide (289/776), which might influence the accuracy of identification in some cases. Lower abundance proteins with higher variance will tend to have lower correlation, hence lower connectivity, potentially obscuring important relationships and excluding lower abundance proteins from modules and impacting power to detect differential correlations.

### Conclusions

This analysis found changes within the CSF protein network in modules and pathways of established relevance to the pathogenesis of ALS, including those linked to the known functions of TDP-43. The diversity of alterations suggests that successful treatment of ALS will require targeting multiple pathways. Restoration of alterations in the CSF protein network might be a useful group-level outcome measure to detect disease modifying effects in therapeutic trials targeting a broad range of potentially pathogenic pathways in ALS.

## Data Availability Statement

Publicly available datasets were analyzed in this study. This data can be found here: https://www.ebi.ac.uk/pride/archive PRIDE archive Accession: PXD024219, DOI: 10.6019/PXD024219.

## Ethics Statement

The studies involving human participants were reviewed and approved by South Central Oxford Ethics Committee B (08/H0605/85) and NRES Central Committee South Central – Berkshire (14/SC/0083 and 10/H0505/71). The participants provided their written informed consent to participate in this study.

## Author Contributions

All authors contributed to study design. AT prepared the manuscript and figures. All authors read and approved the final manuscript.

## Conflict of Interest

MH has received payment for Advisory Board attendance/consultancy for Biogen, Roche, CuraSen Therapeutics, Evidera, Manus Neurodynamica and the MJFF Digital Health Assessment Board. MH is a co-applicant on a patent application related to smartphone predictions in Parkinson’s (PCT/GB2019/052522) pending. The remaining authors declare that the research was conducted in the absence of any commercial or financial relationships that could be construed as a potential conflict of interest.
