# Supplementary material for: Network Analysis of the CSF Proteome Characterizes Convergent Pathways of Cellular Dysfunction in ALS
Source: Front Neurosci. 2021 Mar 17;15:642324. doi: 10.3389/fnins.2021.642324 (PMC8010303; doi:10.3389/fnins.2021.642324)
Supplement: Supplementary file 1 [file Table_1.DOCX]

| **Module** | **GO ID** | **Term** | **Annotated proteins** | **Significant** | **Expected** | **Enrichment** | **p-value** | **FDR-adjusted p-value** | **Ontology** |
| --- | --- | --- | --- | --- | --- | --- | --- | --- | --- |
| 1 | GO:0005634 | nucleus | 175 | 90 | 42.05 | 2.14 | 4.10E-19 | 2.98E-16 | Cellular Component |
|  | GO:0005829 | cytosol | 166 | 77 | 39.88 | 1.93 | 5.30E-13 | 3.85E-10 | Cellular Component |
|  | GO:1903561 | extracellular vesicle | 345 | 118 | 82.89 | 1.42 | 2.10E-09 | 1.52E-06 | Cellular Component |
|  | GO:0032993 | protein-DNA complex | 10 | 10 | 2.4 | 4.17 | 5.30E-07 | 0.00038372 | Cellular Component |
|  | GO:1990904 | ribonucleoprotein complex | 44 | 37 | 10.57 | 3.50 | 4.70E-06 | 0.0033981 | Cellular Component |
|  | GO:0005925 | focal adhesion | 52 | 27 | 12.49 | 2.16 | 5.90E-06 | 0.0042598 | Cellular Component |
|  | GO:0000785 | chromatin | 11 | 11 | 2.64 | 4.17 | 8.90E-06 | 0.0064169 | Cellular Component |
|  | GO:0015630 | microtubule cytoskeleton | 22 | 15 | 5.29 | 2.84 | 3.10E-05 | 0.02232 | Cellular Component |
|  | GO:0099513 | polymeric cytoskeletal fiber | 20 | 14 | 4.81 | 2.91 | 4.00E-05 | 0.02876 | Cellular Component |
|  | GO:0006412 | translation | 45 | 36 | 10.98 | 3.28 | 6.70E-15 | 4.45E-11 | Biological Process |
|  | GO:0019080 | viral gene expression | 26 | 25 | 6.34 | 3.94 | 1.10E-14 | 7.30E-11 | Biological Process |
|  | GO:0006614 | SRP-dependent cotranslational protein ta... | 25 | 24 | 6.1 | 3.93 | 1.20E-14 | 7.96E-11 | Biological Process |
|  | GO:0000184 | nuclear-transcribed mRNA catabolic proce... | 26 | 24 | 6.34 | 3.79 | 1.20E-13 | 7.96E-10 | Biological Process |
|  | GO:0022613 | ribonucleoprotein complex biogenesis | 15 | 15 | 3.66 | 4.10 | 1.20E-07 | 0.0007962 | Biological Process |
|  | GO:0003723 | RNA binding | 89 | 67 | 22.25 | 3.01 | 5.00E-26 | 5.79E-23 | Molecular Function |
|  | GO:0003735 | structural constituent of ribosome | 25 | 24 | 6.25 | 3.84 | 2.10E-14 | 2.43E-11 | Molecular Function |
|  | GO:0045296 | cadherin binding | 30 | 21 | 7.5 | 2.80 | 1.40E-07 | 0.00016184 | Molecular Function |
|  | GO:0032550 | purine ribonucleoside binding | 11 | 10 | 2.75 | 3.64 | 6.80E-06 | 0.007854 | Molecular Function |
|  | GO:0032561 | guanyl ribonucleotide binding | 11 | 10 | 2.75 | 3.64 | 6.80E-06 | 0.007854 | Molecular Function |
|  | GO:0035639 | purine ribonucleoside triphosphate bindi... | 49 | 26 | 12.25 | 2.12 | 1.20E-05 | 0.013836 | Molecular Function |
|  | GO:0019901 | protein kinase binding | 26 | 16 | 6.5 | 2.46 | 6.20E-05 | 0.071424 | Molecular Function |
| 2 | GO:0016021 | integral component of membrane | 185 | 59 | 32.19 | 1.83 | 1.10E-08 | 8.00E-06 | Cellular Component |
|  | GO:0031224 | intrinsic component of membrane | 211 | 71 | 36.72 | 1.93 | 8.90E-06 | 0.0064614 | Cellular Component |
|  | GO:0098982 | GABA-ergic synapse | 9 | 7 | 1.57 | 4.46 | 0.00011 | 0.07975 | Cellular Component |
|  | GO:0061564 | axon development | 66 | 27 | 11.29 | 2.39 | 7.70E-06 | 0.0511203 | Biological Process |
| 4 | GO:0005579 | membrane attack complex | 7 | 6 | 0.75 | 8.00 | 7.90E-06 | 0.0057433 | Cellular Component |
|  | GO:0019835 | cytolysis | 13 | 10 | 1.4 | 7.14 | 4.10E-07 | 0.00272199 | Biological Process |
|  | GO:0004252 | serine-type endopeptidase activity | 20 | 10 | 2.2 | 4.55 | 1.10E-05 | 0.012738 | Molecular Function |
| 5 | GO:0005886 | plasma membrane | 323 | 48 | 25.59 | 1.88 | 4.30E-10 | 3.12E-07 | Cellular Component |
|  | GO:0006958 | complement activation, classical pathway | 69 | 35 | 5.58 | 6.27 | 8.90E-25 | 5.91E-21 | Biological Process |
|  | GO:0002377 | immunoglobulin production | 33 | 23 | 2.67 | 8.61 | 5.00E-20 | 3.32E-16 | Biological Process |
|  | GO:0006897 | endocytosis | 102 | 36 | 8.25 | 4.36 | 8.70E-19 | 5.77E-15 | Biological Process |
|  | GO:0038096 | Fc-gamma receptor signaling pathway invo... | 33 | 20 | 2.67 | 7.49 | 1.60E-15 | 1.06E-11 | Biological Process |
|  | GO:0050853 | B cell receptor signaling pathway | 30 | 19 | 2.43 | 7.82 | 2.90E-15 | 1.92E-11 | Biological Process |
|  | GO:0050871 | positive regulation of B cell activation | 30 | 19 | 2.43 | 7.82 | 2.90E-15 | 1.92E-11 | Biological Process |
|  | GO:0038095 | Fc-epsilon receptor signaling pathway | 29 | 18 | 2.35 | 7.66 | 3.10E-14 | 2.06E-10 | Biological Process |
|  | GO:0042742 | defense response to bacterium | 44 | 21 | 3.56 | 5.90 | 1.80E-13 | 1.19E-09 | Biological Process |
|  | GO:0050900 | leukocyte migration | 65 | 23 | 5.26 | 4.37 | 2.30E-11 | 1.53E-07 | Biological Process |
|  | GO:0002920 | regulation of humoral immune response | 60 | 22 | 4.85 | 4.54 | 4.70E-11 | 3.12E-07 | Biological Process |
|  | GO:0002250 | adaptive immune response | 98 | 43 | 7.93 | 5.42 | 1.90E-06 | 0.0125951 | Biological Process |
|  | GO:0045087 | innate immune response | 100 | 21 | 8.09 | 2.60 | 1.50E-05 | 0.09942 | Biological Process |
|  | GO:0003823 | antigen binding | 50 | 31 | 3.5 | 8.86 | 4.00E-28 | 4.63E-25 | Molecular Function |
|  | GO:0034987 | immunoglobulin receptor binding | 29 | 19 | 2.03 | 9.36 | 4.00E-17 | 4.63E-14 | Molecular Function |
| 6 | GO:0098978 | glutamatergic synapse | 34 | 11 | 3.18 | 3.46 | 0.00011 | 0.07997 | Cellular Component |
| 9 | GO:0005833 | hemoglobin complex | 3 | 3 | 0.03 | 100.00 | 4.60E-07 | 0.00033442 | Cellular Component |
|  | GO:0031838 | haptoglobin-hemoglobin complex | 4 | 3 | 0.04 | 75.00 | 1.80E-06 | 0.0013068 | Cellular Component |
|  | GO:0015701 | bicarbonate transport | 5 | 5 | 0.05 | 100.00 | 1.00E-11 | 6.64E-08 | Biological Process |
|  | GO:0015669 | gas transport | 4 | 4 | 0.04 | 100.00 | 2.60E-09 | 1.73E-05 | Biological Process |
|  | GO:0015893 | drug transport | 9 | 4 | 0.08 | 50.00 | 3.20E-07 | 0.00212384 | Biological Process |
|  | GO:0004089 | carbonate dehydratase activity | 3 | 3 | 0.03 | 100.00 | 5.50E-07 | 0.0006369 | Molecular Function |
|  | GO:0005344 | oxygen carrier activity | 3 | 3 | 0.03 | 100.00 | 5.50E-07 | 0.0006369 | Molecular Function |
|  | GO:0031720 | haptoglobin binding | 3 | 3 | 0.03 | 100.00 | 5.50E-07 | 0.0006369 | Molecular Function |
|  | GO:0019825 | oxygen binding | 4 | 3 | 0.04 | 75.00 | 2.20E-06 | 0.002541 | Molecular Function |
|  | GO:0020037 | heme binding | 6 | 3 | 0.06 | 50.00 | 1.10E-05 | 0.012694 | Molecular Function |
|  | GO:0004601 | peroxidase activity | 8 | 3 | 0.08 | 37.50 | 3.00E-05 | 0.03459 | Molecular Function |
|  | GO:0031721 | hemoglobin alpha binding | 2 | 2 | 0.02 | 100.00 | 7.90E-05 | 0.091008 | Molecular Function |
| 10 | GO:0005577 | fibrinogen complex | 5 | 3 | 0.05 | 60.00 | 4.60E-06 | 0.0033442 | Cellular Component |
|  | GO:0045907 | positive regulation of vasoconstriction | 3 | 3 | 0.03 | 100.00 | 4.90E-07 | 0.00325311 | Biological Process |
|  | GO:1902042 | negative regulation of extrinsic apoptot... | 4 | 3 | 0.04 | 75.00 | 2.00E-06 | 0.013276 | Biological Process |
|  | GO:2000352 | negative regulation of endothelial cell ... | 4 | 3 | 0.04 | 75.00 | 2.00E-06 | 0.013276 | Biological Process |
|  | GO:0034116 | positive regulation of heterotypic cell-... | 5 | 3 | 0.05 | 60.00 | 4.90E-06 | 0.0325164 | Biological Process |
|  | GO:0045921 | positive regulation of exocytosis | 5 | 3 | 0.05 | 60.00 | 4.90E-06 | 0.0325164 | Biological Process |
|  | GO:0090277 | positive regulation of peptide hormone s... | 5 | 3 | 0.05 | 60.00 | 4.90E-06 | 0.0325164 | Biological Process |
|  | GO:1900026 | positive regulation of substrate adhesio... | 6 | 3 | 0.06 | 50.00 | 9.70E-06 | 0.0643401 | Biological Process |

**Supplementary table 1** ***Gene ontology enrichment analysis for individual modules*** (FDR-adjusted p < 0.10).

| **Protein 1** | **HGNC symbol 1** | **Protein 2** | **HGNC symbol 2** | ***r*, Healthy control** | ***r*, disease** | **FDR-adjusted *p*** | **Disease** |
| --- | --- | --- | --- | --- | --- | --- | --- |
| E5RG81 | CA1 | P02008 | HBZ | -0.60 | 0.69 | 0.057 | ALS |
| O14556 | GAPDHS | P14174 | MIF | 0.93 | 0.25 | 0.064 | ALS |
| Q5JP53 | TUBB | P68104 | EEF1A1 | 0.97 | 0.58 | 0.035 | ALS |
| P06899 | H2BC11 | Q5JP53 | TUBB | 0.97 | 0.57 | 0.057 | ALS |
| Q96S96 | PEBP4 | Q16849 | PTPRN | 0.80 | -0.28 | 0.057 | ALS |
| H0YD17 | CD44 | Q16849 | PTPRN | 0.85 | -0.36 | 0.006 | ALS |
| D6RAX3 | PCDH1 | V9GYE3 | APOA2 | -0.93 | -0.21 | 0.035 | ALS |
| H0YN26 | ANP32A | H0YCG2 | LAMP2 | -0.68 | 0.51 | 0.057 | ALS |
| H0Y449 | YBX1 | P01814 | IGHV2-70 | 0.92 | -0.26 | 0.057 | ALS |
| E5RGR6 | GFRA2 | F5GZQ4 | LDHA | -0.80 | 0.26 | 0.064 | ALS |
| F8W785 | GOLIM4 | J3KSV6 | ALDOC | -0.99 | 0.30 | 0.035 | ALS |
| C9JRG0 | HBD | P02008 | HBZ | 0.51 | 0.98 | 0.06 | PD |
| E5RG81 | CA1 | P02008 | HBZ | -0.60 | 0.86 | 0.017 | PD |
| P32119 | PRDX2 | P02008 | HBZ | -0.28 | 0.86 | 0.067 | PD |
| P32119 | PRDX2 | P00918 | CA2 | -0.48 | 0.79 | 0.067 | PD |
| K7EQQ3 | KRT9 | M0R3G9 | PLD3 | -0.65 | 0.65 | 0.095 | PD |
| K7ES70 | MFAP4 | Q5T848 | GPR158 | -0.61 | 0.71 | 0.067 | PD |
| K7EKH5 | ALDOC | Q5T848 | GPR158 | 0.61 | -0.69 | 0.095 | PD |
| A0A087X0S5 | COL6A1 | H0YDE5 | KIAA1549L | -0.71 | 0.64 | 0.06 | PD |
| A0A0J9YXX1 | IGHV5-10-1 | P01303 | NPY | -0.74 | 0.53 | 0.1 | PD |
| P07195 | LDHB | A0A087WTY6 | NBL1 | 0.71 | -0.57 | 0.095 | PD |
| H7C4W4 | FSTL1 | A0A087WTY6 | NBL1 | -0.30 | 0.86 | 0.067 | PD |
| H7C4W4 | FSTL1 | H0YLF3 | B2M | 0.30 | -0.87 | 0.067 | PD |
| P07195 | LDHB | C9JMK5 | PIK3IP1 | 0.78 | -0.54 | 0.06 | PD |
| P07195 | LDHB | M0R1M6 | UBA52 | 0.69 | -0.65 | 0.067 | PD |
| H0Y4H1 | PTPRF | O75752 | B3GALNT1 | -0.46 | 0.83 | 0.095 | PD |
| P07195 | LDHB | E5RFR1 | PENK | 0.61 | -0.68 | 0.095 | PD |
| P01768 | IGHV3-30 | O75882 | ATRN | 0.75 | -0.52 | 0.095 | PD |
| G3V5M2 | PNP | P07195 | LDHB | 0.50 | -0.78 | 0.067 | PD |
| G3V4U0 | FBLN5 | H7C4W4 | FSTL1 | 0.37 | -0.87 | 0.046 | PD |
| E9PNW4 | AL049629.2 | G3V4U0 | FBLN5 | 0.46 | -0.85 | 0.038 | PD |
| A0A087X253 | AP2B1 | G3V4U0 | FBLN5 | 0.22 | -0.86 | 0.097 | PD |
| G3V5M2 | PNP | G3V4U0 | FBLN5 | 0.64 | -0.85 | 0.001 | PD |
| A0A087X253 | AP2B1 | Q8TBP5 | FAM174A | -0.46 | 0.79 | 0.085 | PD |
| Q6UXB8 | PI16 | A0A075B6K5 | IGLV3-9 | 0.85 | -0.26 | 0.095 | PD |
| A0A087X0S5 | COL6A1 | E9PNW4 | AL049629.2 | -0.63 | 0.71 | 0.067 | PD |
| Q8NGR9 | OR1N2 | E9PHK0 | CLEC3B | 0.74 | -0.79 | 0.06 | PD |
| Q8TER0 | SNED1 | A0A087WXX2 | ALDOB | -0.42 | 0.89 | 0.097 | PD |

**Supplementary table 2** ***Differential correlation analysis significant protein pairs*** (FDR-adjusted p < 0.10). ALS – amyotrophic lateral sclerosis; PD – Parkinson’s disease.

| **Uniprot** | **HGNC symbol** | **Gradient** | **p-value** | **FDR-adjusted p-value** |
| --- | --- | --- | --- | --- |
| P02750 | LRG1 | 0.010 | <0.001 | 0.018 |
| Q96IY4 | CPB2 | 0.008 | 0.003 | 0.097 |
| C9JRT3 | cDNA | 0.006 | <0.001 | 0.032 |
| P05543 | SERPINA7 | 0.006 | 0.003 | 0.097 |
| P10643 | C7 | 0.005 | 0.001 | 0.079 |
| K7EKQ5 | LGALS3BP | 0.008 | 0.001 | 0.079 |
| P08727 | KRT19 | 0.013 | 0.002 | 0.096 |
| P01861 | IGHG4 | 0.007 | 0.002 | 0.079 |
| A0A087WX80 | LAMA2 | 0.027 | 0.002 | 0.089 |
| B5MCV4 | C1S | 0.006 | 0.002 | 0.079 |
| Q96JF0 | ST6GAL2 | -0.012 | <0.001 | 0.018 |
| H0Y9I4 | PAM | -0.010 | <0.001 | 0.026 |
| O95445 | APOM | -0.011 | 0.003 | 0.098 |
| H7C4X7 | SCG5 | -0.008 | <0.001 | 0.018 |
| Q9BQT9 | CLSTN3 | -0.013 | 0.003 | 0.099 |
| Q06481 | APLP2 | -0.007 | <0.001 | 0.024 |
| Q16849 | PTPRN | -0.010 | 0.001 | 0.079 |
| X6RKN2 | NFASC | -0.010 | 0.001 | 0.079 |
| H3BMF9 | SEMA7A | -0.008 | 0.001 | 0.074 |
| O75326 | SEMA7A | -0.007 | 0.002 | 0.079 |
| Q9UM22 | EPDR1 | -0.026 | <0.001 | 0.026 |
| Q5T8H6 | CNTFR | -0.012 | 0.002 | 0.079 |
| F5H658 | DHX8 | -0.020 | 0.003 | 0.097 |
| A0A087WWT2 | NRN1 | -0.019 | <0.001 | 0.018 |
| E7EPF1 | ADAM22 | -0.007 | 0.001 | 0.079 |

**Supplementary table 3** ***Longitudinally varying proteins in ALS patients*** (FDR-adjusted p < 0.10).


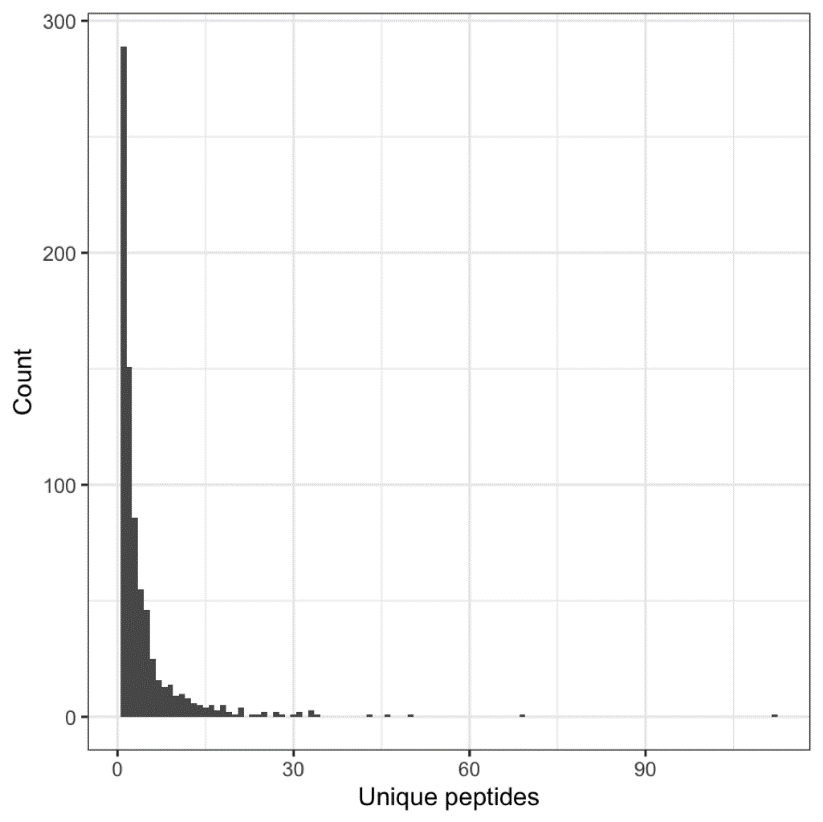


**Supplementary figure 1** Number of unique peptides for proteins included in network analysis.


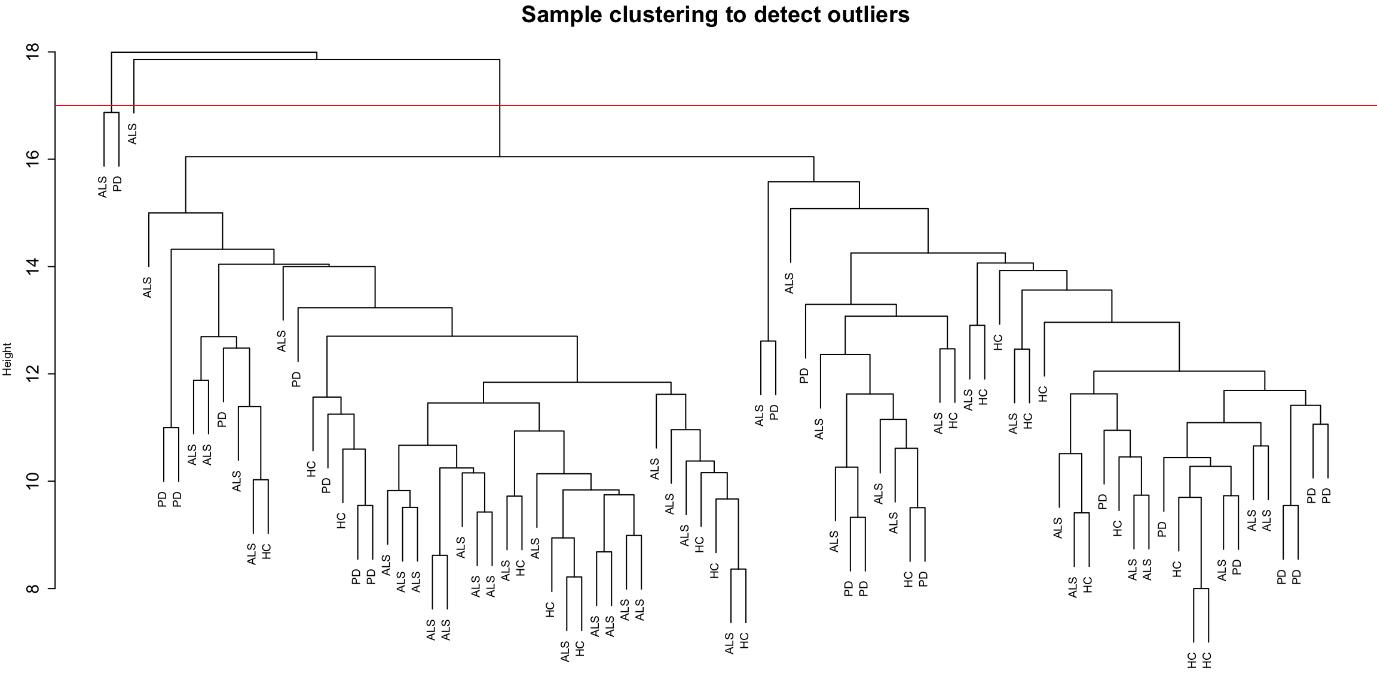


**Supplementary Figure 2.** Hierarchical clustering identified three outlying samples (2 ALS, 1 PD) which were excluded from subsequent analysis. ALS – amyotrophic lateral sclerosis; PD – Parkinson’s disease.


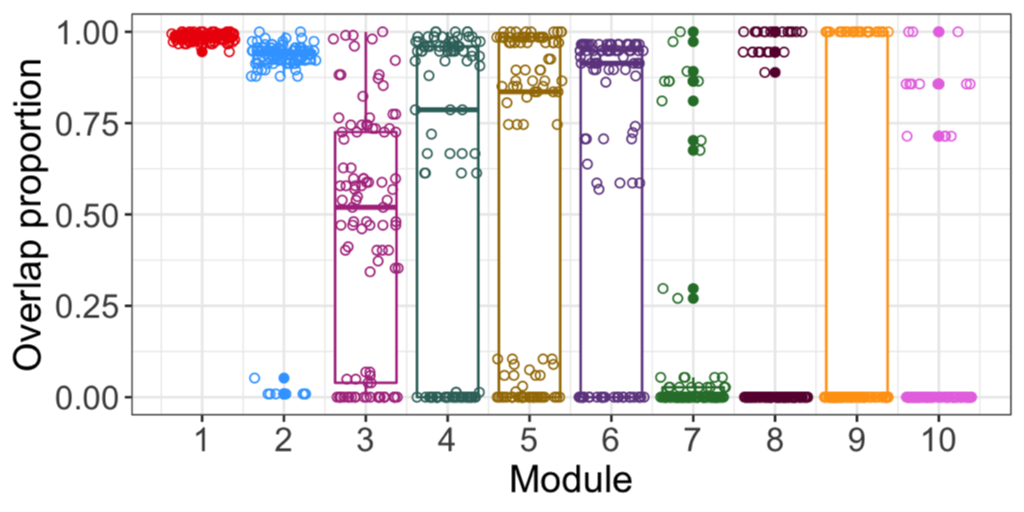


**Supplementary Figure 3** Module membership stability by iterating network construction, randomly leaving out one sample each iteration and comparing module assignment with the reference network.
